# Oenin and Quercetin Copigmentation: Highlights From Density Functional Theory

**DOI:** 10.3389/fchem.2018.00245

**Published:** 2018-06-28

**Authors:** Yunkui Li, Mario Prejanò, Marirosa Toscano, Nino Russo

**Affiliations:** ^1^College of Enology, Northwest A&F University, Yangling, China; ^2^Dipartimento di Chimica e Tecnologie Chimiche, Università della Calabria, Arcavacata di Rende, Italy

**Keywords:** copigmentation, oenin, quercetin, hydrogen bonding, density functional theory, anthocyanin, red wine, copigment

## Abstract

Making use of anthocyanin copigmentation, it is possible to effectively improve color quality and stability of red wines and other foods. This can be done by selecting strong copigments, but a 1-fold experimental screening usually entails a high cost and a low efficiency. The aim of this work is to show how a theoretical model based on density functional theory can be useful for an accurate and rapid prediction of copigmentation ability of a copigment. The present study, concerning the copigmentation between oenin and quercetin under the framework of implicit solvent, indicates that, in these conditions, the intermolecular hydrogen bonds play an important role in the system stabilization. The dispersion interaction slightly affects the structure, energies and UV-Vis spectral properties of the copigmentation complex.

## Introduction

Red wine color depends mainly on composition of anthocyanins (Han et al., 2015) which possess a skeleton of flavylium cation being prone to reactions of proton transfer or hydration, resulting in the decline of wine color (Fulcrand et al., 2006; Escribano-Bailon and Santos-Buelga, 2012; Trouillas et al., 2015, 2016). This problem is also present in other foods containing anthocyanins as natural colorants (Trouillas et al., 2016; Cortez et al., 2017). However, it is an inherent nature of anthocyanins to associate with copigments (Escribano-Bailon and Santos-Buelga, 2012; Trouillas et al., 2015, 2016; Cortez et al., 2017; Qian et al., 2017; Gras et al., 2018), usually colorless phenolic compounds, which help them to maintain the flavylium cation state and thus to get the color stabilized (Boulton, 2001; Gómez-Míguez et al., 2006; Malaj et al., 2013; Trouillas et al., 2016; Qian et al., 2017; Gras et al., 2018). This is known as copigmentation effect, featured with red-shift of anthocyanin spectra and enhancement of color stability. Since copigmentation contributes 30~50% to the total color of young red wines (Boulton, 2001; Gómez-Míguez et al., 2006; Lambert et al., 2011; Han et al., 2015), it is a good way to improve color quality of red wines. Current researches mainly focus on copigmentation mechanism (Di Meo et al., 2012; Kalisz et al., 2013; Trouillas et al., 2015; Zhang et al., 2016), physicochemical factors and their optimization (Lambert et al., 2011; Malaj et al., 2013; Heras-Roger et al., 2016; Zhang et al., 2016) and structural features of anthocyanins and copigments (Kunsági-Máté et al., 2006; Lambert et al., 2011; Malaj et al., 2013; Teixeira et al., 2013; Zhang et al., 2016) with the aim to strengthen copigmentation. The selection of strong copigments from large samples shall be a potential approach to reinforce copigmentation. However, a 1-fold experimental screening usually entails a high cost and a low efficiency. Experiments usually choose a small number of copigments (Boulton, 2001; Gómez-Míguez et al., 2006; Kunsági-Máté et al., 2006; Lambert et al., 2011; Kalisz et al., 2013 Malaj et al., 2013; Teixeira et al., 2013; Xu et al., 2015; Zhang et al., 2016), but it is difficult to individuate the best one.

Different from experiments, a quantum mechanical (QM) screening allows to get structures and properties of copigmentation systems (Quartarolo and Russo, 2011; Di Meo et al., 2012; Kalisz et al., 2013; Rustioni et al., 2013; Trouillas et al., 2015, 2016), such as binding energies and spectral shifts, in a quicker, time-saving and money-saving way. This implies the use of a reliable theoretical model which consists in a good strategy for searching the most stable conformers of copigmentation complex in the conformational space, in an appropriate choice of the QM methods for geometry optimization, energetic and spectral calculations and in a reliable description of solvation effects (Li et al., 2011a,b; Nave et al., 2012; Rustioni et al., 2013; Trouillas et al., 2015, 2016; Marpaung et al., 2017). Actually, finding out the most stable conformer can be done by calculations totally based on a QM approach, starting from few most probable orientations, or by a preliminary molecular dynamics (MD) simulation followed by a QM refinement (Di Meo et al., 2012; Trouillas et al., 2015, 2016). The former way is expected to be more reliable and more time-saving than the latter one as long as reasonable QM methods are utilized and appropriate initial guess of orientations are adopted (Trouillas et al., 2016).

The nature of the non-covalent interaction between anthocyanin and copigment is not quite clear and the suggested π-π stacking configuration may be driven by dispersion forces, hydrogen bonds (HBs), hydrophobic effects, etc (Dimitrić Marković et al., 2005; Kunsági-Máté et al., 2006; Di Meo et al., 2012; Kalisz et al., 2013; Teixeira et al., 2013; Trouillas et al., 2015, 2016; Zhang et al., 2016). This complexity calls to consider various possibilities for the initial guess, and a careful selection of computational methods. In some previous reports, anthocyanin glycone was substituted by a methyl group in order to explore the general nature of copigmentation and reduce computational cost (Di Meo et al., 2012; Trouillas et al., 2015, 2016). However, it is quite reasonable to take the holo glycoside into account when we screen strong copigments against a specific anthocyanin. The hydroxyl-rich sugar moiety ought to have notable impact.

The purpose of this paper is to investigate the copigmentation process between malvidin-3-*O*-glucoside (oenin), normally the highest content anthocyanin in *Vitis vinifera* young red wines and a determining factor of wine color (Han et al., 2015), and quercetin (see Scheme 1 in Supporting Information), a representative and intensively studied copigment in red wines (Lambert et al., 2011). Our QM study includes conformer search strategy, methods for geometry optimization, energy evaluation, spectral shift and solvation effects estimation.

**Scheme 1:**
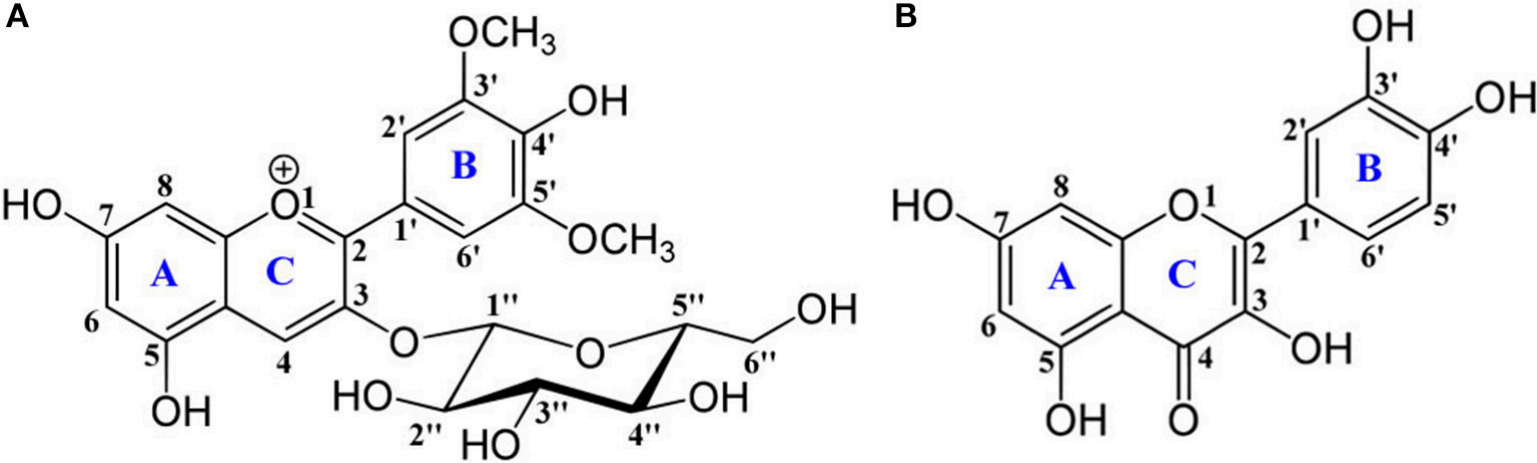
Chemical structures of **(A)** oenin and **(B)** quercetin, of which the backbone atoms and rings are numbered.

## Methods and computational details

According to previous researches based on density functional theory (DFT) (Grimme et al., 2010; Anouar et al., 2012; Di Meo et al., 2012; Trouillas et al., 2015, 2016), the hybrid B3LYP-D3 functional combined with the 6-31+G(d) basis set with polarization and diffusion functions on heavy atoms was adopted for geometry optimization of individual oenin and individual quercetin, as well as the conformer search of oenin/quercetin complex. Vibrational frequency calculations were performed with the same method to verify that each obtained structure is a minimum on the potential surface, and to get corrections for thermodynamic functions. Integral equation formalism polarizable continuum model (IEFPCM) was used to describe water solvent effect in all calculations except spectral evaluation (Li et al., 2011a,b; Trouillas et al., 2015, 2016).

The binding energies for selected conformers were obtained according to Equation 1:

(1) $$\begin{array}{r} \Delta E_{\text{binding}}=E_{\text{complex}}-\underset{i}{\sum }E_{i} \end{array}$$

where *i* stands for oenin or quercetin. The geometries of the complex conformers, oenin and quercetin were optimized individually. In order to explore the impact of functional and basis set on the binding energy, benchmark single point computations on previously optimized geometries were performed with normal hybrid GGA functionals B3LYP-D3(BJ) and B3LYP-D3, range-separated hybrid functionals ωB97X-D and CAM-B3LYP-D3, and meta-GGA functional M06-2X-D3, each of which was combined with 6-311++G(d,p), aug-cc-pVDZ, aug-cc-pVTZ, and aug-cc-pVQZ basis sets. The basis set superposition error (BSSE) (Simon et al., 1996) of complex energy was assessed by counterpoise method and calculated with CAM-B3LYP-D3/aug-cc-pVTZ.

Binding energy is the sum of total distortion energy (Equation 2) of oenin and quercetin and their interaction energy (Equation 3) (Scaranto et al., 2011):

(2) $$\begin{array}{r} \Delta E_{\text{dist}}=\underset{i}{\sum }\left(E_{i}{,}_{\text{complexed}}-E_{i}\right) \end{array}$$

(3) $$\begin{array}{r} \Delta E_{\text{inter}}=E_{\text{complex}}-\underset{i}{\sum }E_{\text{i}}{,}_{\text{complexed}} \end{array}$$

where “complexed” means the geometry of oenin or quercetin in the complex. Thus, the binding, distortion and interaction energies for 12 selected conformers are estimated. The Boltzmann distribution of the nine most stable conformers was evaluated according to relative Gibbs free energies, compared with the free energy of the optimal conformer.

The dispersion contribution to binding and interaction energies of the 12 conformers were calculated with CAM-B3LYP-D3/aug-cc-pVTZ. To explore the CT character, the electronic population analysis was achieved by CHelpG formalism (Breneman and Wiberg, 1990) with CAM-B3LYP-D3/aug-cc-pVTZ for ground states and TD-ωB97X-D/cc-pVDZ for excited states. Pearson correlation analysis was made by IBM SPSS Statistics to investigate the relation between energies and CT.

Functionals, with different HF exchange component, of B3LYP, PBE0, B3PW91, CAM-B3LYP, ωB97X-D, and M06-2X coupled with cc-pVDZ basis set and state-specific PCM (SS-PCM) were employed to predict the spectral shift of the optimal conformer (Yanai et al., 2004; Chai and Head-Gordon, 2008; Quartarolo and Russo, 2011; Di Meo et al., 2012; Trouillas et al., 2015, 2016). One of the best-performed functional, ωB97X-D, was further used to evaluate the spectral shift of other 11 conformers. All calculations were achieved with Gaussian09 packages (Frisch et al., 2009).

The visualization of the non-covalent contributions were obtained by using the NCIPLOT software version 3.0 (Johnson et al., 2010; Contreras-Garcia et al., 2011).

## Results and discussion

Twelve most probable orientations of complex oenin/quercetin were considered (see Figure S1). Orientations 1~4 show the backbone of quercetin being parallel or antiparallel to that of oenin, like largely suggested by previous studies (Kunsági-Máté et al., 2006; Di Meo et al., 2012; Kalisz et al., 2013; Teixeira et al., 2013; Trouillas et al., 2015, 2016). This interaction mode should favor a great extent of electron delocalization between oenin and quercetin, with a good chance of getting a stable conformer. Intuitively, orientations 1 and 2 are beneficial for dispersion interaction and charge transfer (CT), namely the apparent π-π stacking, while orientations 3 and 4 are also favorable for HB interactions between quercetin and hydroxyl-rich sugar moiety of oenin. Since HB interactions could play a key role, more orientations offering this possibility were taken into examination. In orientations 5~8, quercetin takes a triangular-shaped arrangement to simultaneously interact with the sugar part of oenin mainly by HB, and with backbone of oenin mainly by HB or dispersion force. In orientations 9~12, quercetin exhibits as a sandwiched layer located between sugar and backbone segments of oenin, which allows similar or weaker interactions as in the triangular-shaped arrangement.

### Structural feature

**Figure 1:**
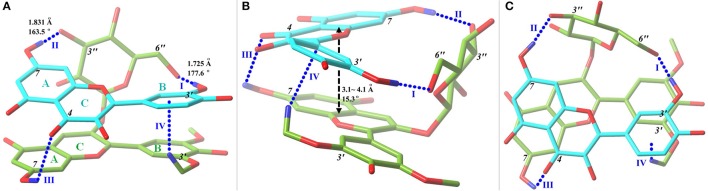
Front **(A)**, side **(B)**, and top **(C)** views of the optimal conformer **5** with a tube molecular representation. Carbon atoms are colored in green for oenin and in cyan for quercetin. Oxygen and hydrogen (involved in hydrogen bonds) atoms are depicted in red and blue, respectively. HBs, presented in blue dashed lines and numbered, are exhibited with key parameters. Parameters for III are 2.439 Å and 113.6°, while for IV, the C-H…π, parameters are 2.590 Å and 149.5°. The stacking distance and dihedral between AC-rings of oenin and AC-rings of quercetin are also annotated. A fogging depth-cueing is used to improve perception.

Beginning from the most probable orientations (Figure S1), nine most stable conformers **1**~**9** were obtained by scanning the complex conformational space. Other three higher-energy conformers **10**~**12** were also selected to comparatively explore the structural feature of copigmentation (see Table 1). Orthographic views of the optimal conformer **5** were depicted in Figure 1, while views of remaining ones were illustrated in Figure S2. π-π stacking, namely ring-stacking (see Table 1), phenomenon is universal for all stable conformers (Di Meo et al., 2012). The stacking form includes translated parallel (**10**, **12**), also called “parallel-displaced” (Di Meo et al., 2012), rotated parallel (**1**, **4**, **5**, **9**), antiparallel (**2**, **3**, **6**, **7**, **8**, **11**) and aslant parallel (**2**, **5**, **8**, **10**, **11**, **12**) that has a stacking dihedral bigger than 10°. Among them, rotated and aslant parallel forms are rarely reported before. The distance and dihedral for antiparallel stacking rings are 3.3~3.4 Å and 0.5~7.0°, respectively, while for aslant parallel stacking rings are 3.0~4.1 Å and 12.1~27.9°, respectively. The absent (**1**, **3**, **6**, **11**), displaced (**1**, **2**, **3**, **4**, **6**, **8**, **9**, **11**), or aslant (**2**, **8**, **10**, **11**, **12**) stacking of any ring tends to reduce the strength of stacking, and thus reduce the stability of the complex.

**Table 1:** Main parameters for HB and ring-stacking (RS) and relative Gibbs free energies Δ*G*_relative_ for the examined conformers.

**Conformer**	**HB (D-H⋯A)^a^**			**RS**			**Δ*G*_relative_^e^**
	**Quantity**	***r* (H⋯A)^b^**	**∠(D-H⋯A)^c^**	**Type (Oenin⋯Quercetin)^d^**	***r*(RS)^b^**	**∠(RS)^c^**	
**1**	1	1.851	169.4	AC⋯AC	3.3	4.6	4.67
**2**	1	1.858	165.3	AC⋯B, B⋯AC	3.6, 3.3	18.7, 1.5	3.96
**3**	2	1.730	164.5	AC⋯AC	3.3	3.0	4.92
**4**	1	1.745	172.3	AC⋯A, B⋯B	3.3, 3.4	1.0, 6.6	3.65
**5**	4	1.725	177.6	AC⋯AC	3.1~4.1	15.3	0.00
**6**	2	1.731	164.5	AC⋯AC	3.3	3.0	4.94
**7**	2	1.730	164.7	AC⋯AC	3.3	1.6	2.75
**8**	3	1.902	170.8	AC⋯B, B⋯AC	3.4~4.0, 3.3	12.1, 7.0	4.95
**9**	1	1.745	172.3	AC⋯A, B⋯B	3.3, 3.4	0.5, 6.6	3.48
**10**	3	1.835	139.7	AC⋯AC, B⋯B	3.3, 3.2~4.1	0.5, 22.8	7.03
**11**	2	1.852	153.3	A⋯B	3.0~4.1	27.9	8.55
**12**	3	1.845	138.5	AC⋯AC, B⋯B	3.3, 3.2~4.1	0.6, 22.9	6.88

*Distance in Å, angle in degree and energy in kcal/mol*.

a *Parameters are shown only for the strongest hydrogen bond, while others are shown in Figure 1 and Figure S2. D and A stand for hydrogen donor and acceptor, respectively. The HB type is O-H…O for all conformers, except that conformer **5** has another weak C-H…π*.

b *Distance between H and hydrogen acceptor, or between two rings*.

c *Angle of a hydrogen bond, or dihedral of two rings*.

d *A, B and AC denote the A-ring, B-ring and AC-rings, respectively*.

e *The energy of conformer **5** was taken as the reference*.

**Figure 2:**
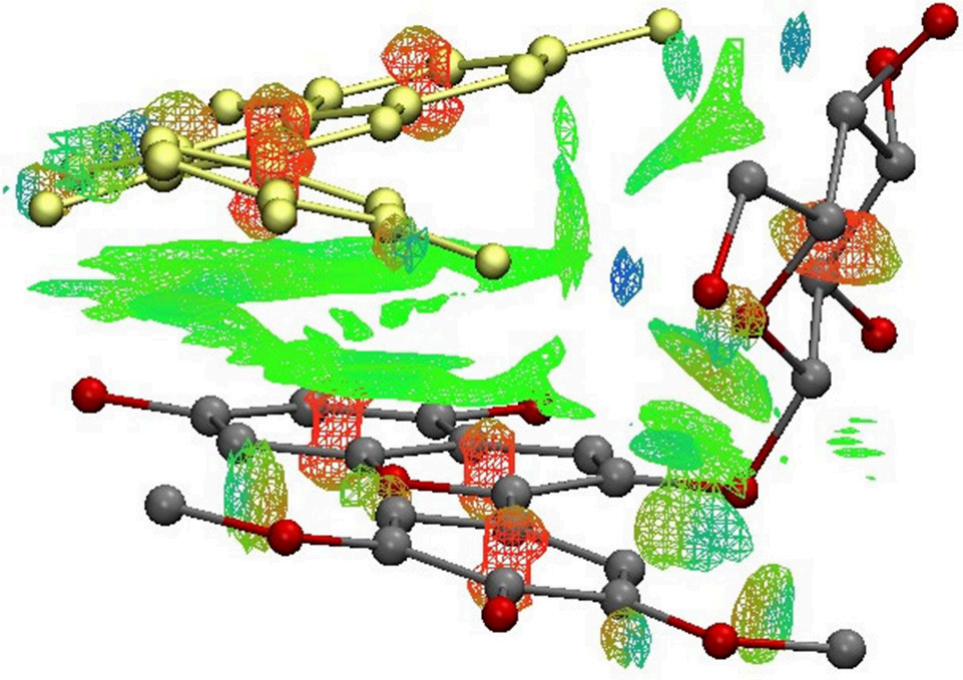
Visualization of non-covalent interactions (Green = weack interactions, Red = repulsive interactions, Blue-red = attractive interactions) in complex **5**.

More importantly, the HB effect is critical to copigmentation complex stability, which has not been highlighted with substantial evidence before (Di Meo et al., 2012; Kalisz et al., 2013; Zhang et al., 2016). Complex **5** is the most stable one although it does not presents the B…B stacking and has a shifted and inclined AC…AC stacking. Its stability could be attributed to two strong HBs connecting the *6*″-OH of oenin with *3*′-OH of quercetin and *3*″-OH of oenin with *7*-OH of quercetin, and another weak HB between *7*-OH of oenin and *4*-OH of quercetin. In addition, a weak interaction between *3*′-OCH_3_ of oenin with conjugate π-electrons of B-ring of quercetin is observed. In Figure 2, different kinds of non-covalent interactions in complex 5 are indicated. Other than HBs it is possible to note the presence of many van der Walls contributions between the two moieties. So, also these interactions contribute to stabilize the complex.

Similar situation happens in conformers **1**~**3** and **6**~**9**, in which, however, there is insufficient ring overlap. On the contrary, in spite of a good AC…AC stacking, **10** and **12** have higher relative energies (Table 1), which should be partly ascribed to weak HBs formed between oenin and quercetin. Oenin *3*″-OH group is most likely to form a strong HB, and *6*″-OH and *3*′-OCH_3_ may also have great possibility. For quercetin, *3*′-OH has an absolute advantage to form a strong HB. Thus, a substitution of any of these groups shall dramatically affect copigmentation stability. As in our case, another recent study found a great influence of HB on the structure of catechol dimer (Barone et al., 2017). Besides, it's worth noting that the glycone of anthocyanin can have a double-effect. On one hand it may enhance the interaction between pigment and copigment via its hydroxyls to form HBs, on the other hand, its steric effect may impede copigment approaching the skeleton of anthocyanin, as uncovered by experiments (Gras et al., 2018).

A great structural distortion takes place when oenin and quercetin associates with each other. Table 2 shows that the total torsion angle between AC-rings and B-ring is 23.0 ± 7.4° for all conformers, including 7.7 ± 4.7° distortion from oenin and 15.3 ± 10.7° from quercetin. This mainly comes from the flexibility of glucoside and B-ring, and results in the formation of strong HBs. Thus, intermolecular HBs, causing significant structural distortion of the two monomers, shall further affect the stability and spectra properties of the copigmentation complex.

**Table 2:** Inter-ring torsion angle between AC-rings and B-ring (in degree)*^a^*.

**Conformer**	**∠AC-B (Oenin)**			**∠AC-B (Quercetin)**			**Total torsion**
	**Individual**	**Complexed**	**Torsion**	**Individual**	**Complexed**	**Torsion**	
**1**	16.3	15.7	0.6	8.0	24.1	16.1	16.7
**2**	16.3	6.2	10.1	8.0	25.0	17.0	27.1
**3**	16.3	4.3	12.0	8.0	13.0	5.0	17.0
**4**	16.3	5.8	10.5	8.0	4.0	4.0	14.5
**5**	16.3	11.4	4.9	8.0	28.5	20.5	25.4
**6**	16.3	4.2	12.1	8.0	13.0	5.0	17.1
**7**	16.3	2.8	13.5	8.0	14.5	6.5	20.0
**8**	16.3	5.6	10.7	8.0	23.3	15.3	26.0
**9**	16.3	6.1	10.2	8.0	4.0	4.0	14.2
**10**	16.3	13.5	2.8	8.0	36.3	28.3	31.1
**11**	16.3	14.9	1.4	8.0	42.6	34.6	36.0
**12**	16.3	12.8	3.5	8.0	35.4	27.4	30.9
Mean±S.D.			7.7 ± 4.7			15.3 ± 10.7	23.0 ± 7.4

a *The angle torsion is defined as the angle difference between the complexed state and individual state*.

It is noteworthy that this study is based on the implicit solvent model (Li et al., 2011a,b). An explicit description of the solvent (Trouillas et al., 2015, 2016) could modify the obtained results in that it may change the hydrogen pattern and the relative stability of the conformers.

### Energetic behavior

Binding energy is a key index for evaluation of copigmentation ability of a copigment. The binding energy values of conformer **5** estimated with different functionals and basis sets are listed in Table 3. All the methods predict a prominent binding energy, in agreement with experimental observations (Lambert et al., 2011) and other calculations (Di Meo et al., 2012). The impact of functional is not significant, even if CAM-B3LYP-D3 seems better according to experimental binding enthalpy. For all tested functionals, a larger basis set provides a lower binding energy, implying the necessity of employing high-level basis set to capture the complicated interactions between oenin and quercetin as comprehensively as possible. Still, 6-311++G(d,p) and aug-cc-pVDZ basis sets are good enough to give reasonable results.

**Table 3:** Binding energies of conformer **5** with different functionals and basis sets (in kcal/mol)*^a^*.

**Functionals**	**6-311++G(d,p)*^b^***	**aug-cc-pVDZ*^c^***	**aug-cc-pVTZ*^d^***	**aug-cc-pVQZ*^d^***
B3LYP-D3(BJ)	−24.81	−25.26	−26.20	−25.35
B3LYP-D3	−24.51	−24.95	−25.89	−25.04
ωB97X-D	−24.97	−25.32	−26.00	−24.64
CAM-B3LYP-D3	−23.88	−24.19	−24.89	−24.04
M06-2X-D3	−23.96	−24.41	−24.95	

a *Geometry is based on B3LYP-D3/6-31+G(d). Thermal corrections were computed at B3LYP-D3/6-31+G(d) level, while the total electronic energies were calculated with corresponding functionals and basis sets*.

b *Counterpoise BSSEs were estimated by corresponding functionals combined with 6-311++G(d,p)*.

c *Counterpoise BSSEs were estimated by CAM-B3LYP-D3/aug-cc-pVDZ*.

d *Counterpoise BSSEs were estimated by CAM-B3LYP-D3/aug-cc-pVTZ*.

Actually, binding energy consists of two parts: distortion of copigmentation system and interaction between anthocyanin and copigment (Scaranto et al., 2011). It is easy to confuse binding energy and interaction energy, of which the latter one really represents the stability of both the complex and the color. The more negative the interaction energy, the more stable the complex and the color. Thus, interaction energy should be taken as the index for copigmentation ability evaluation of a copigment. CAM-B3LYP-D3/ aug-cc-pVTZ approach was used to evaluate the binding, distortion and interaction energies for 12 selected conformers, and the Boltzmann weights (BW) of the nine most stable conformers (see Table 4). As can be seen from values of BW, conformer **5** has overwhelming possibility to exist in real wine solution, due to strong HBs formed between oenin and quercetin as described before. This indicates that neglecting the sugar moiety of oenin, or miss-capturing the optimal conformer assisted by HBs may lead to a higher binding energy, which is much closer to the experimental value but not rational. Noteworthy distortion energies of 0.41~5.11 kcal/mol for conformers **1**~**9** are obtained, in accordance with the structural distortion. This suggests that the use of interaction energy rather than binding energy is more appropriate to describe copigmentation stability.

**Table 4:** Binding energies Δ*E*_binding_, distortion energies Δ*E*_dist_, interaction energies Δ*E*_inter_, and Boltzmann weights *BW* (in kcal/mol)*^a^*.

**Conformer**	**Δ*E*_binding_**	**Δ*E*_dist_**	**Δ*E*_inter_**	***BW* (%)**
**1**	−16.23	1.30	−17.52	0.04
**2**	−17.97	0.41	−18.38	0.12
**3**	−18.33	3.67	−22.00	0.02
**4**	−19.53	1.13	−20.66	0.21
**5**	−24.89	1.34	−26.23	98.34
**6**	−18.32	3.66	−21.42	0.02
**7**	−21.37	1.39	−21.98	0.94
**8**	−18.64	5.11	−21.25	0.02
**9**	−19.55	1.13	−22.75	0.27
**10**	−15.98	5.44	−23.75	
**11**	−14.67	6.58	−21.95	
**12**	−16.02	5.93	−20.68	

a *Geometry is based on B3LYP-D3/6-31+G(d). Thermal corrections were computed at B3LYP-D3/6-31+G(d) level, while the total electronic energies and counterpoise BSSEs were calculated with CAM-B3LYP-D3/aug-cc-pVTZ*.

Dispersion contribution to binding and interaction energies, as well as intermolecular CT of ground and excited states for 12 conformers are displayed in Table 5. The dispersive interaction contributes −22.01 ± 0.79 kcal/mol to the binding energy and −22.41 ± 0.85 kcal/mol to the interaction energy. This manifests that the difference of dispersion contribution between conformers or between binding and interaction energies is indistinctive. Since the dispersion contribution is of the same magnitude as the binding or interaction energy itself for each conformer, as previous studies revealed (Kunsági-Máté et al., 2006; Di Meo et al., 2012; Teixeira et al., 2013), one may conclude that the dispersion plays an uppermost contribution to copigmentation. Nevertheless, a Pearson correlation analysis shows that the correlation coefficient between dispersion energy and binding energy is 0.348 (*p* = 0.268), while it is 0.440 (*p* = 0.153) between dispersion contribution and interaction energy (see Table 6). This means that the dispersion contribution is significant to neither binding nor interaction energy, just as a similar finding in catechol dimer (Barone et al., 2017). The most likely scenario is that HBs play a more important role to binding or interaction energy, and thus to stability of copigmentation (Kalisz et al., 2013; Zhang et al., 2016; Barone et al., 2017). This is supported by the former structural analysis. To elucidate this issue, a reliable interaction energy decomposition is necessary in a future study.

**Table 5:** Dispersion contribution to binding Δ*E*_disp,binding_ and interaction Δ*E*_dist,inter_ energies, intermolecular CT of ground *q*_GS_ and excited *q*_ES_ states for the 12 conformers (energies in kcal/mol, charge in |e|) *^a^*.

**Conformer**	**Δ*E*_disp,binding_**	**Δ*E*_disp,inter_**	***q*_GS_*^b^***	***q*_ES_*^b^***
**1**	−20.82	−21.56	−0.07	0.71
**2**	−20.90	−21.37	−0.04	0.75
**3**	−22.86	−23.03	−0.02	0.70
**4**	−21.92	−22.18	−0.06	−0.03
**5**	−21.76	−21.93	−0.06	−0.06
**6**	−22.87	−23.04	−0.02	0.70
**7**	−23.30	−23.57	0.00	0.69
**8**	−22.78	−23.28	0.08	0.72
**9**	−21.90	−22.15	−0.06	−0.03
**10**	−21.80	−23.01	−0.02	0.11
**11**	−21.45	−20.89	0.08	0.07
**12**	−21.73	−22.93	−0.01	0.13
mean±S.D.	−22.01 ± 0.79	−22.41 ± 0.85	−0.02 ± 0.05	0.37 ± 0.36

a *Geometry is based on B3LYP-D3/6-31+G(d). Dispersion energies and q_GS_ were calculated with CAM-B3LYP-D3/aug-cc-pVTZ. q_ES_ were computed by TD-ωB97X-D/cc-pVDZ. Eelectronic population analysis was achieved by CHelpG formalism*.

b *“−” means the electron is transferred from quercetin to oenin, vice versa*.

**Table 6:** Pearson correlation analysis between energies and CT*^a^*.

	**Δ*E*_binding_**	**Δ*E*_dist_**	**Δ*E*_inter_**	***G*_relative_**	**Δ*E*_disp,binding_**	**Δ*E*_disp,inter_**	***q*_GS_**
Δ*E*_binding_	1	0.605^*^	0.623^*^	0.944^**^	0.348	0.199	0.358
Δ*E*_dist_		1	−0.246	0.808^**^	−0.157	−0.201	0.740^**^
Δ*E*_inter_			1	0.356	0.577^*^	0.440	−0.290
*G*_relative_				1	0.151	0.071	0.560
Δ*E*_disp,binding_					1	0.830^**^	−0.327
Δ*E*_disp,inter_						1	−0.138
*q*_GS_							1

a Superscripts of significance (two-tailed)

** and * *stand for highly significant (p < 0.01) and significant (p < 0.05), respectively*.

### Spectral shift

UV-Vis spectral shift is another key index for giving insight to the copigmentation process. The more red-shift of the spectra, the stronger the copigment. The maximum absorption wavelengths, λ_max_, of oenin and conformer **5**, as well as the experimental and predicted spectral shifts estimated with different functionals are listed in Table 7. All functionals underestimate λ_max_ of oenin and conformer **5** compared with experiments (Lambert et al., 2011), as it happens in previous DFT studies (Di Meo et al., 2012; Trouillas et al., 2015, 2016). However, it is found that range-separated hybrid functionals CAM-B3LYP and ωB97X-D, and meta-GGA functional M06-2X seem to better describe the electronic excitation of copigmentation complex (Di Meo et al., 2012; Trouillas et al., 2015, 2016), since the bathochromic spectral shifts from 7.2 to 9.4 nm are much closer to the experimental value of 8.5 nm (Lambert et al., 2011). Previous evaluation of the spectral shift of 3-*O*-methylcyanidin complexed with quercetin by ωB97X-D also gave reasonable results (Di Meo et al., 2012). Thus, TD-ωB97X-D/cc-pVDZ combined with SS-PCM solvent model is trustworthy to describe the electronic transition and corresponding spectral shifts of copigmentation complex of oenin and quercetin.

**Table 7:** Impact of functionals on spectral shift of conformer **5** (λ_max_ in nm)*^a^*.

**Functional**	**λ_max_(Oenin)**	**λ_max_(Complex)**	**Δλ_max_**	
			**This work**	**expt*^b^***
B3LYP-D3	492.4	493.2	0.8	8.5
CAM-B3LYP-D3	430.1	439.0	8.8	
M062X-D3	429.9	439.2	9.4	
ωB97X-D	424.2	431.4	7.2	
B3PW91-D3	493.5	492.5	−1.0	
PBE0-D3	471.8	476.0	4.2	

a *Geometry is based on B3LYP-D3/6-31+G(d). Spectra were calculated with SS-PCM, TD-DFT-D3/cc-pVDZ*.

b *Lambert et al. (2011)*.

Vertical excitation energies, maximum absorption wavelengths and corresponding shifts, oscillator strengths and molecular orbital (MO) contributions for 12 conformers are exhibited in Table 8. Compared to the spectrum of oenin, each conformer presents an evident bathochromic shift in the range 3.8~14.5 nm, except a slight and notable hypochromatic shift of 0.7 nm and 8.7 nm for conformers **8** and **11**, respectively. Each conformer also has a high oscillator strength. There is a significant dependency (correlation coefficient = 0.781, *p* = 0.003) between the extent of shift and the charge transfer degree in the ground state. A CT from quercetin to oenin (shown in Table 5) can cause a bathochromic shift. More fundamentally, the CT of equilibrium ground state is determined by multiple factors, of which the HB effect rather than the dispersive interaction should be the key, as discussed above under the circumstance of implicit solvent scheme.

**Table 8:** Vertical excitation energies *E*_max_, maximum absorption wavelengths λ_max_, spectral shifts Δλ_max_, oscillator strengths *f* and MO descriptions (%) of 12 conformers (energy in eV, wavelength in nm)*^a^*.

**Conformer**	***E*_max_**	**λ_max_**	**Δλ_max_*^b^***	***f***	**MO**
**1**	2.86	433.4	9.2	0.4545	HOMO-1 → LUMO (78.2)
**2**	2.88	430.3	6.1	0.4364	HOMO-1 → LUMO (84.6)
**3**	2.87	431.7	7.5	0.4602	HOMO-1 → LUMO (82.3)
**4**	2.83	437.7	13.5	0.3807	HOMO-1 → LUMO (79.3)
**5**	2.87	431.4	7.2	0.4157	HOMO-1 → LUMO (81.7)
**6**	2.87	431.7	7.5	0.4601	HOMO-1 → LUMO (82.3)
**7**	2.90	428.0	3.8	0.4461	HOMO-1 → LUMO (80.1)
**8**	2.93	423.4	−0.7	0.3826	HOMO-1 → LUMO (81.1)
**9**	2.83	437.6	13.5	0.3806	HOMO-1 → LUMO (79.3)
**10**	2.83	438.3	14.1	0.2960	HOMO-1 → LUMO (74.3)
**11**	2.98	415.5	−8.7	0.4210	HOMO-1 → LUMO (79.2)
**12**	2.83	438.7	14.5	0.2798	HOMO-1 → LUMO (71.5)

a *Geometry is based on B3LYP-D3/6-31+G(d). The spectra were calculated with SS-PCM, TD-ωB97X-D/cc-pVDZ*.

b *Compared to λ_max_(oenin)*.

**Figure 3:**
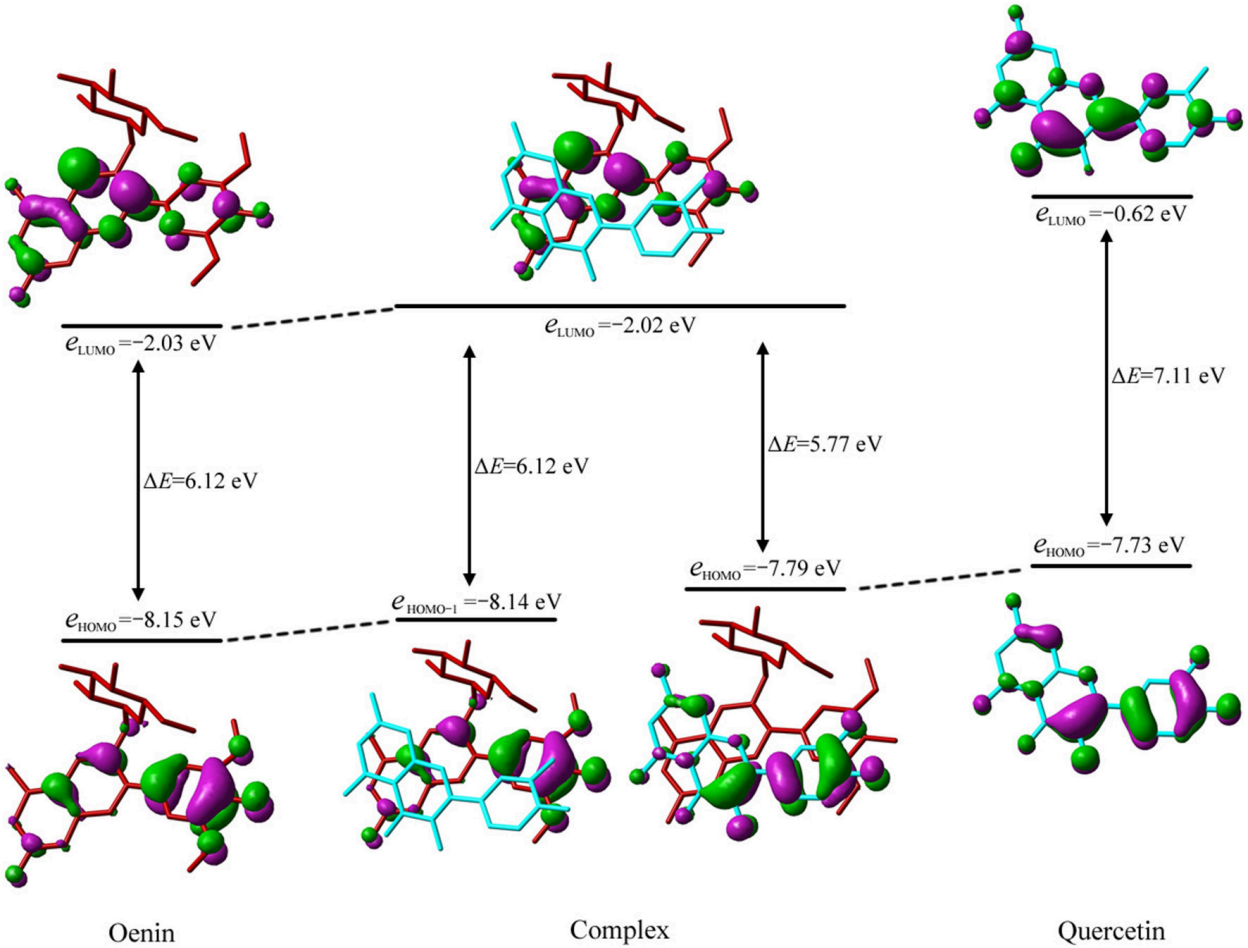
Molecular orbital correlation diagram of oenin, conformer **5** and quercetin. A tube molecular representation was adopted for oenin in red and quercetin in cyan.

For all conformers, the maximum absorptions is due to the transition from the second highest occupied molecular orbital (HOMO-1) to the lowest unoccupied MO (LUMO). MO correlation of oenin, conformer **5** and quercetin (see Figure 3), shows that HOMO, HOMO-1 and LUMO of conformer **5** are close to the HOMO of quercetin, and to the HOMO and LUMO of oenin. The HOMO → LUMO energy gap of the complex is only 5.77 eV, much lower than the gap of quercetin (7.11 eV), and very similar to the gap of oenin. HOMO, HOMO-1, and LUMO of the complex locate mainly on B-ring and C-ring of quercetin, B-ring and AC-rings of oenin. Thus, the HOMO → LUMO transition corresponds to an intermolecular CT from quercetin to oenin (Di Meo et al., 2012; Trouillas et al., 2015, 2016), while HOMO-1 → LUMO refers to an intramolecular CT of oenin. Considering the structure of conformer **5** (Figure 1), B-ring of quercetin almost has no superposition with oenin, and C-ring of quercetin is aslant and translated parallel to AC-rings of oenin with a dihedral of 15.3°. This consistently decreases the overlap between HOMO and LUMO, and thus diminishes the HOMO → LUMO contribution to the maximum absorption of conformer **5**, while protrudes the HOMO-1 → LUMO contribution. Such an intramolecular excitation does not have an apparent intermolecular CT (see Table 5). However, for conformers **1**, **2**, **3**, **6**, **7**, **8**, **10**, and **12**, HOMO-1 → LUMO transition corresponds to an intermolecular excitation that has a quite big CT (Di Meo et al., 2012; Trouillas et al., 2015, 2016).

## Conclusions

In summary, copigmentation of oenin with quercetin was carefully investigated by using density functional theory. From our study the following conclusions can be drawn:

– Among the 12 considered initial copigmentation orientations, 10 are characterized by HB interactions between quercetin and glucoside of oenin. Quercetin assumes a (anti)parallel, triangular-shaped, or sandwiched form located between sugar and backbone segments of oenin. The above 10 orientations include the most stable conformer, which is stabilized by two strong HBs and other two weak interactions. Thus, the intermolecular HB can be considered the driving force of copigmentation process between oenin and quercetin when implicit solvent effect is considered. But it may be different if specific water molecules are taking into account.
– Based on our results it appears that a reliable description of the copigmentation process should comprise the sugar moiety of anthocyanin to allow to take into account the different HB interactions that can occur between hydroxyl-rich glycone of oenin and quercetin.
– Grimme dispersion correction are mandatory to describe dispersive interaction between pigment and copigment.
– CAM-B3LYP-D3 in conjuction with IEFPCM and 6-311++G(d,p) or aug-cc-pVDZ basis set is good enough to provide reasonable binding and interaction energies.
– TD-ωB97X-D/cc-pVDZ partnered with SS-PCM is able to afford trustworthy description of the electronic spectral properties of the copigmentation complex.

The strategy which we have used in this case could further be applied to estimate other copigmentation systems. In this way, it will be possible to screen strong copigments from large-scale samples efficiently, and sequentially improve color quality and stability of red wines and other foods.

## Author contributions

All authors listed have made a substantial contribution to the work and approved its publication. YL designed the protocol, made calculations and wrote the paper. MP made calculations. MT and NR designed the protocol and wrote the paper.

### Conflict of interest statement

The authors declare that the research was conducted in the absence of any commercial or financial relationships that could be construed as a potential conflict of interest.
