# Supplementary material for: Oenin and Quercetin Copigmentation: Highlights From Density Functional Theory
Source: Front Chem. 2018 Jun 28;6:245. doi: 10.3389/fchem.2018.00245 (PMC6031711; doi:10.3389/fchem.2018.00245)
Supplement: Supplementary file 1 [file Data_Sheet_1.DOCX]

***Supplementary Material***

**Density Functional Theory Description of Oenin and Quercetin Copigmentation: A Highlight of Hydrogen Bonding Effect**

**Yunkui Li^*^ , Mario Prejano, Marirosa Toscano, Nino Russo^*^**

*** Correspondence:** Yunkui Li: ykli@nwsuaf.edu.cn; Nino Russo: nino.russo@unical.it

**1 Supplementary Figures**


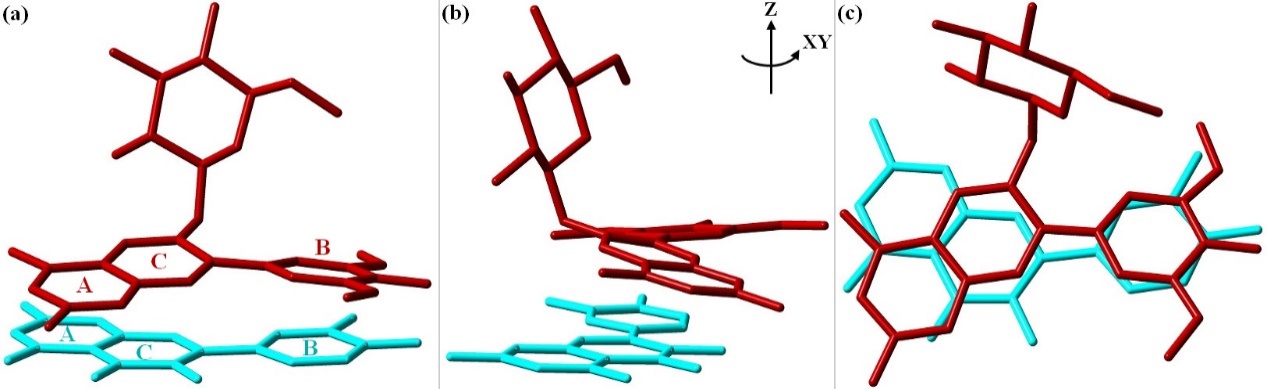


**Figure S1-1**. Orientation 1: orthographic views of a rotated parallel orientation. (a), (b) and (c) stand for the front view, side view and top view, respectively. The label on the top right corner of (b) shows that the potential energy curves along Z-direction and in XY-plane at the minimum of Z-direction shall be scanned in tandem, similarly hereinafter


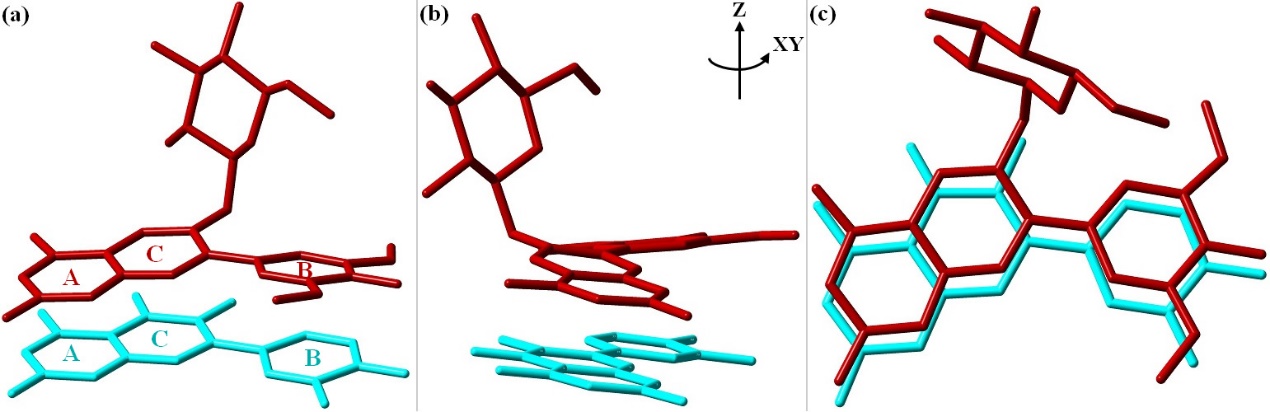


**Figure S1-2**. Orientation 2: orthographic views of a translated parallel orientation


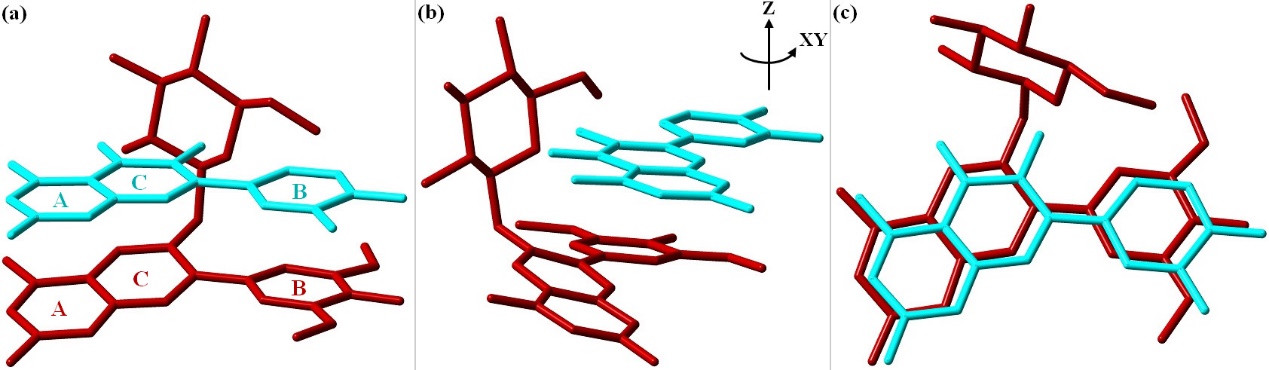


**Figure S1-3**. Orientation 3: orthographic views of a translated parallel orientation


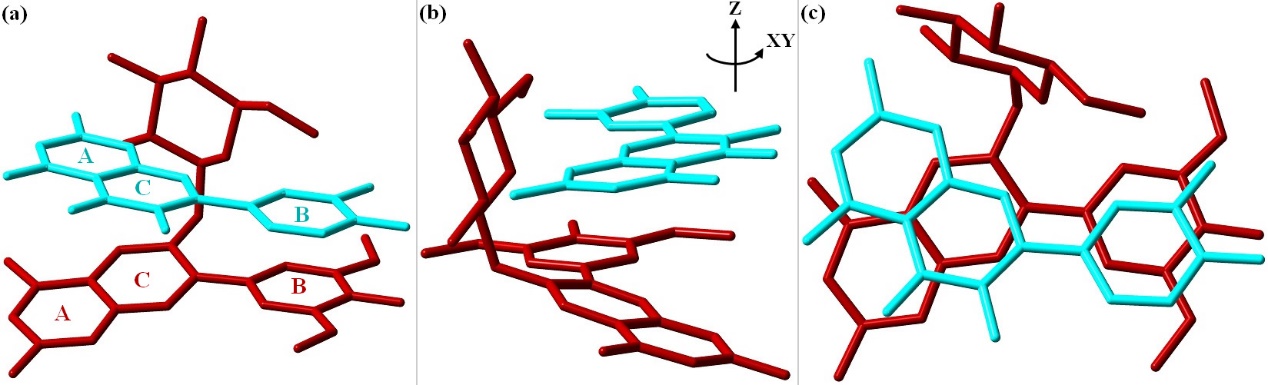


**Figure S1-4**. Orientation 4: orthographic views of a rotated parallel orientation


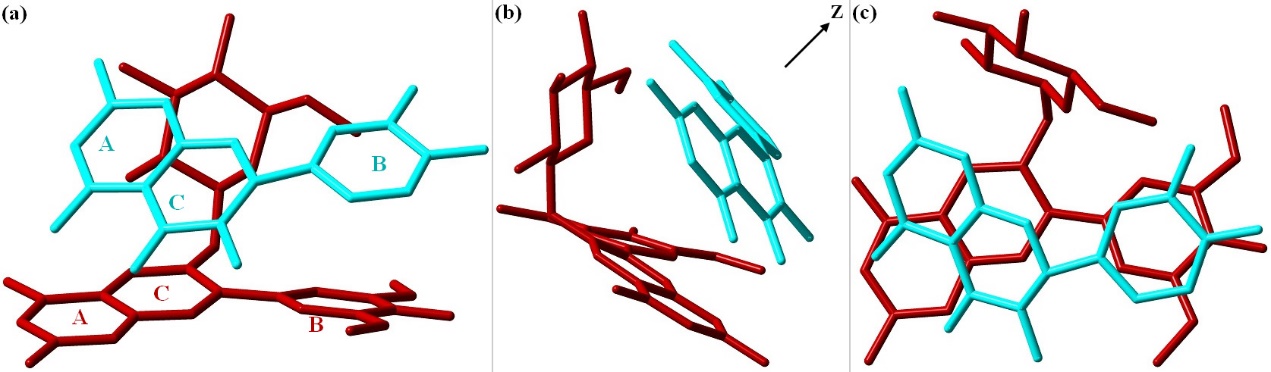


**Figure S1-5**. Orientation 5: orthographic views of an aslant parallel orientation


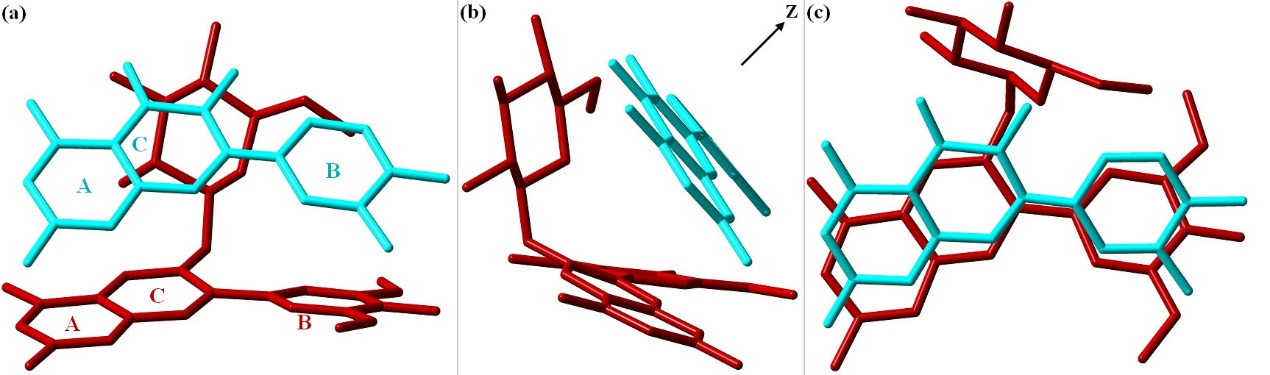


**Figure S1-6**. Orientation 6: orthographic views of an aslant parallel orientation


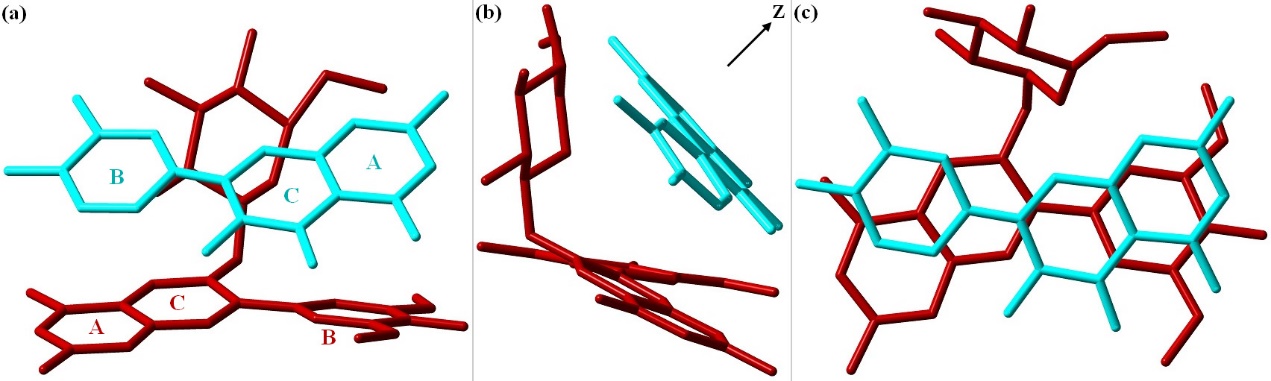


**Figure S1-7**. Orientation 7: orthographic views of an aslant antiparallel orientation


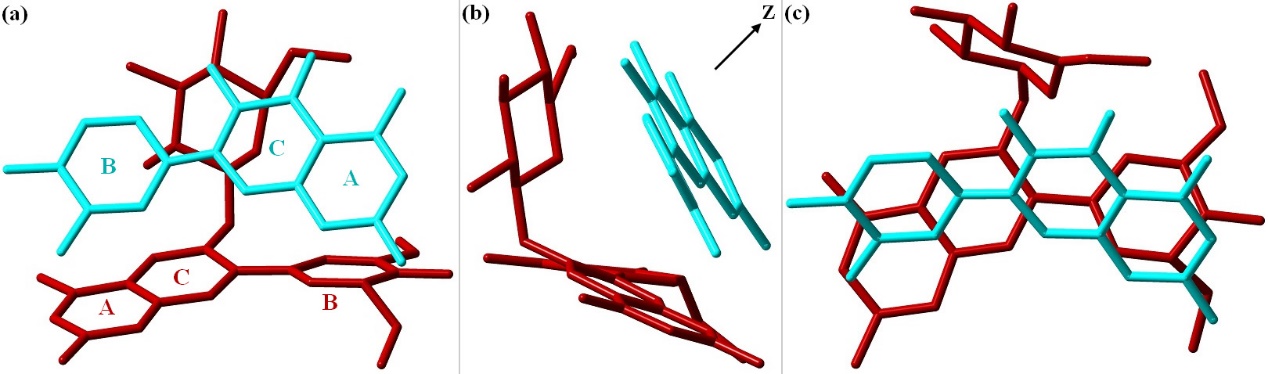


**Figure S1-8**. Orientation 8: orthographic views of an aslant antiparallel orientation


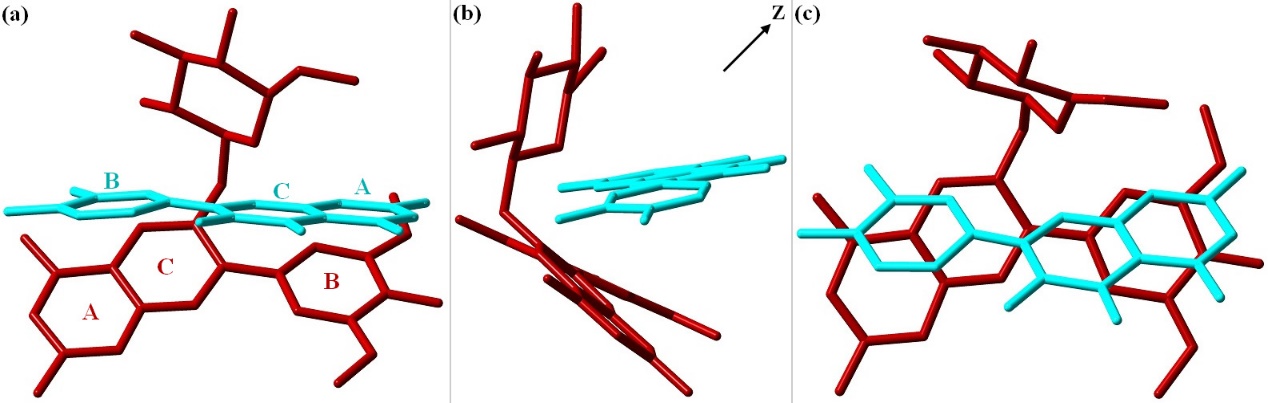


**Figure S1-9**. Orientation 9: orthographic views of an aslant antiparallel orientation


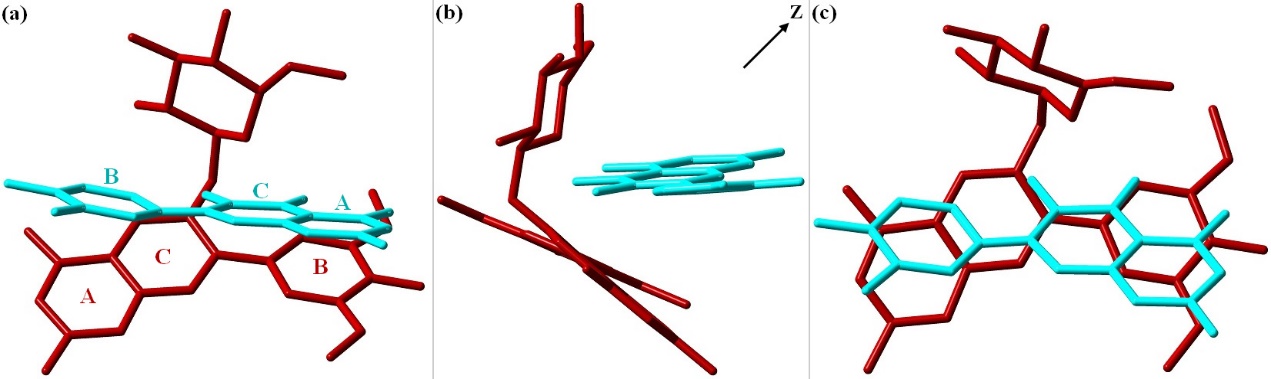


**Figure S1-10**. Orientation 10: orthographic views of an aslant antiparallel orientation


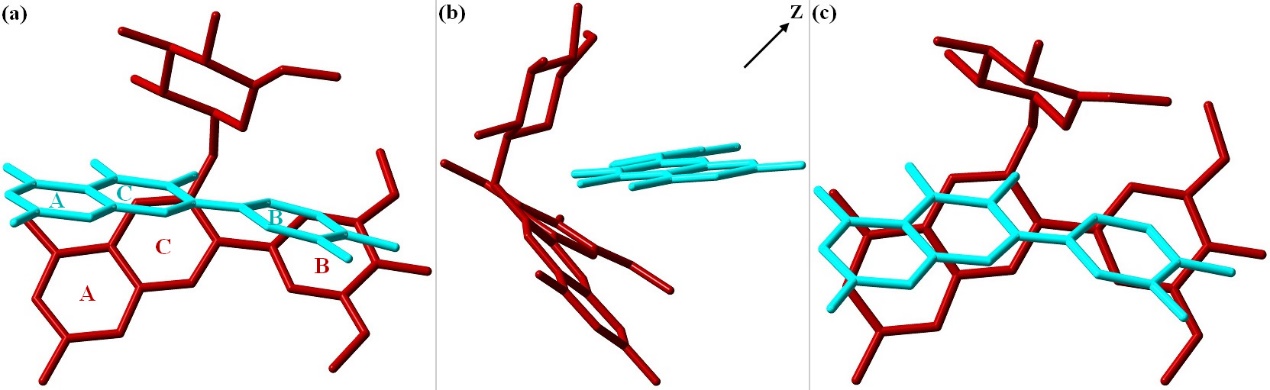


**Figure S1-11**. Orientation 11: orthographic views of an aslant parallel orientation


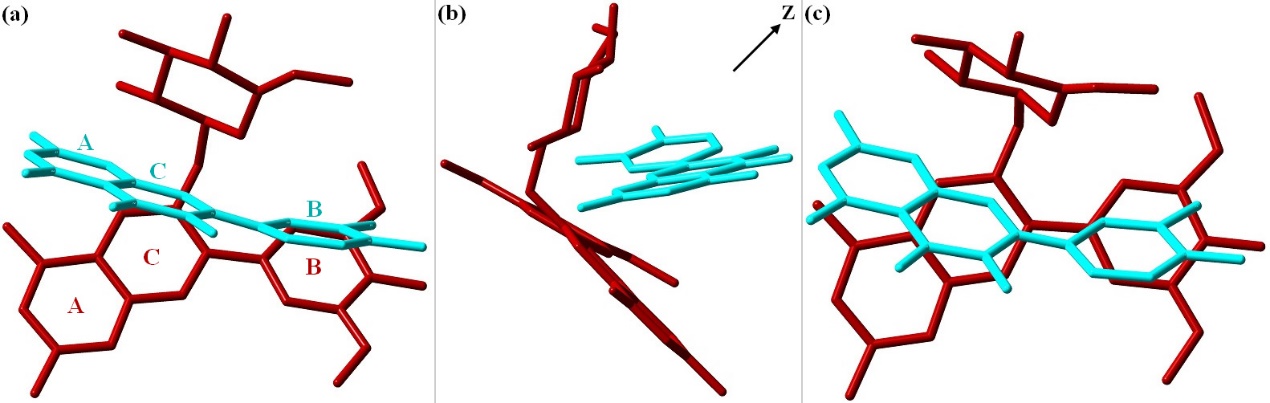


**Figure S1-12**. Orientation 12: orthographic views of an aslant parallel orientation


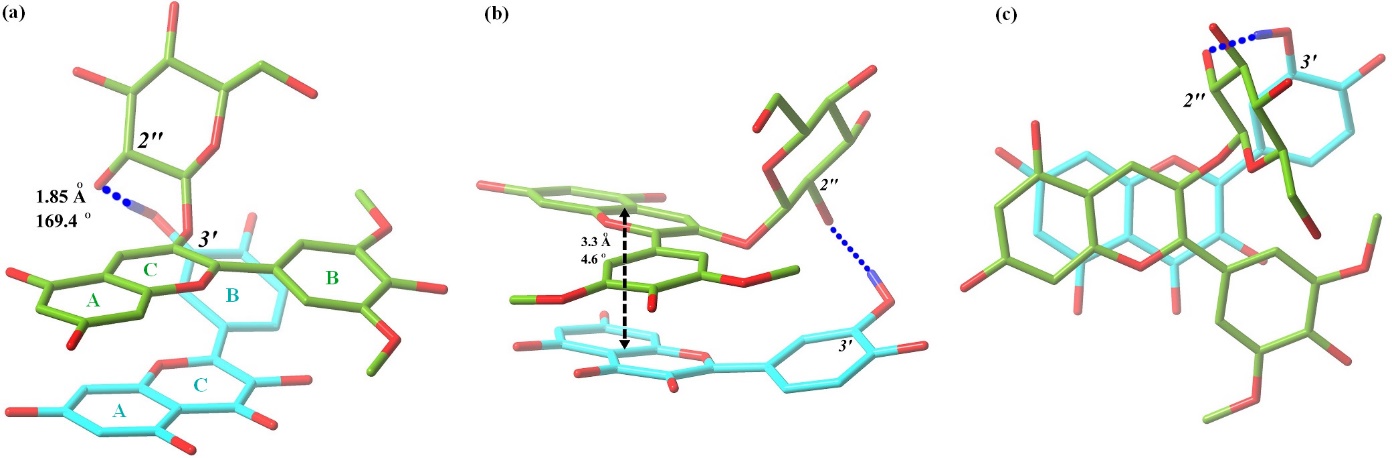


**Figure S2-1**. Front (a), side (b) and top (c) views of conformer **1**


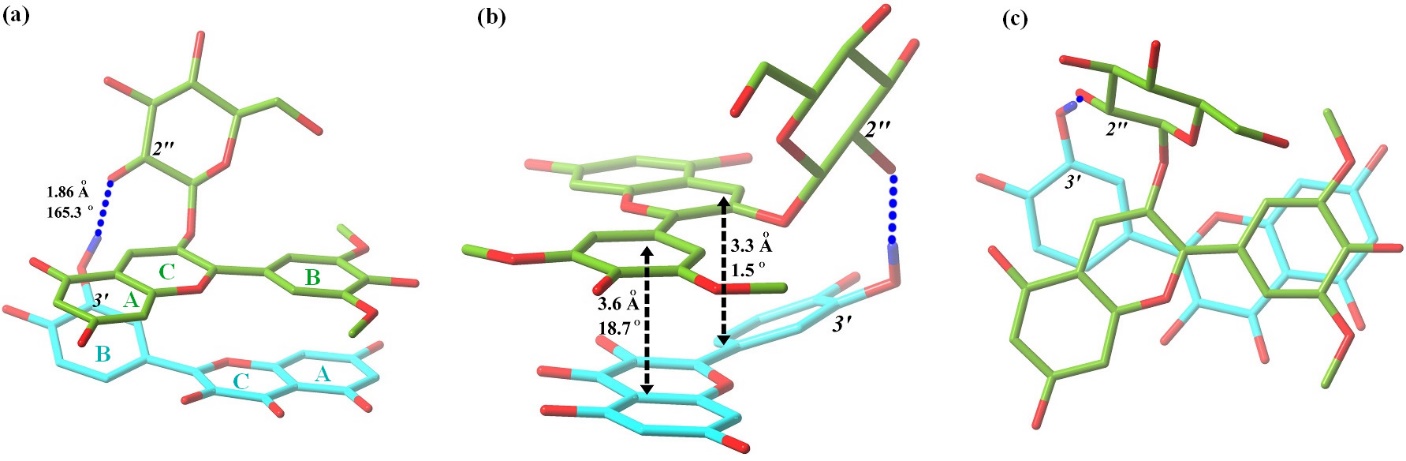


**Figure S2-2**. Front (a), side (b) and top (c) views of conformer **2**


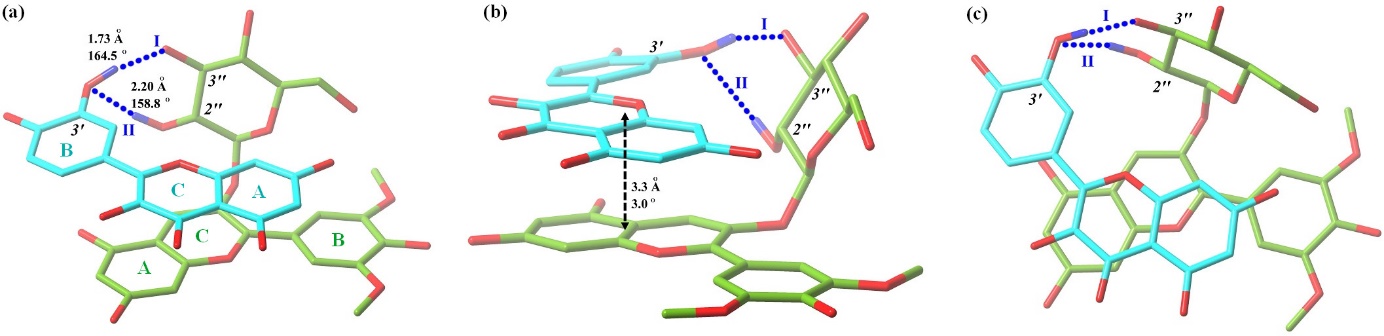


**Figure S2-3**. Front (a), side (b) and top (c) views of conformer **3**


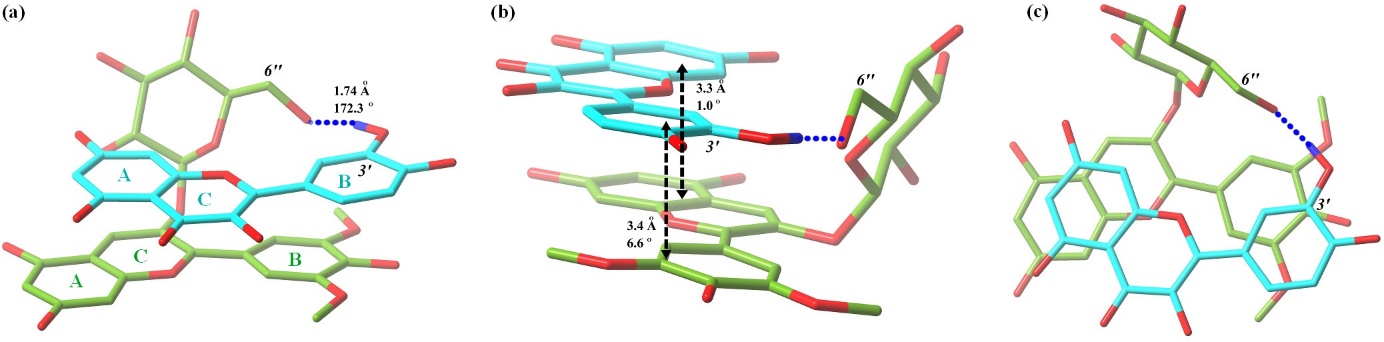


**Figure S2-4**. Front (a), side (b) and top (c) views of conformer **4**


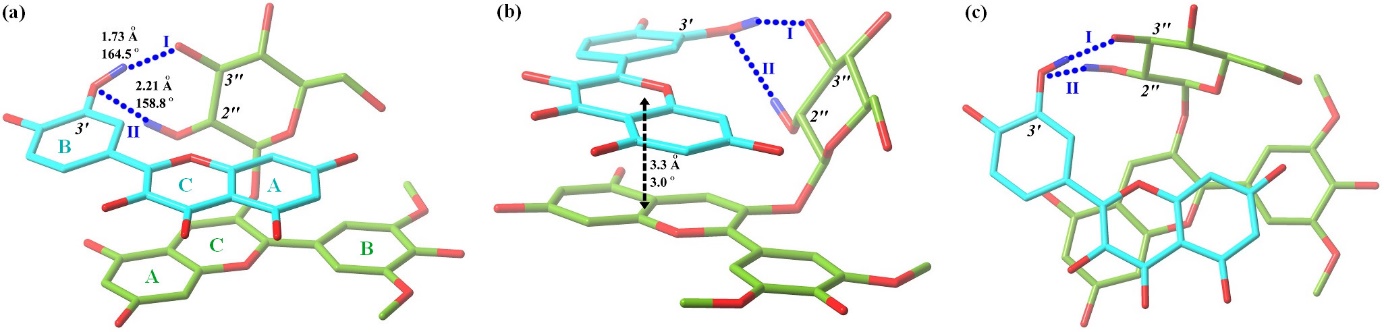


**Figure S2-5**. Front (a), side (b) and top (c) views of conformer **6**


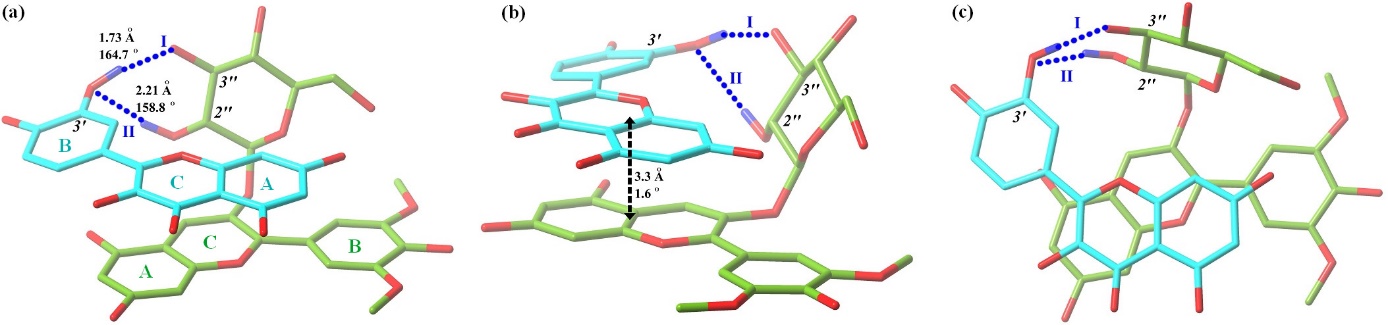


**Figure S2-6**. Front (a), side (b) and top (c) views of conformer **7**


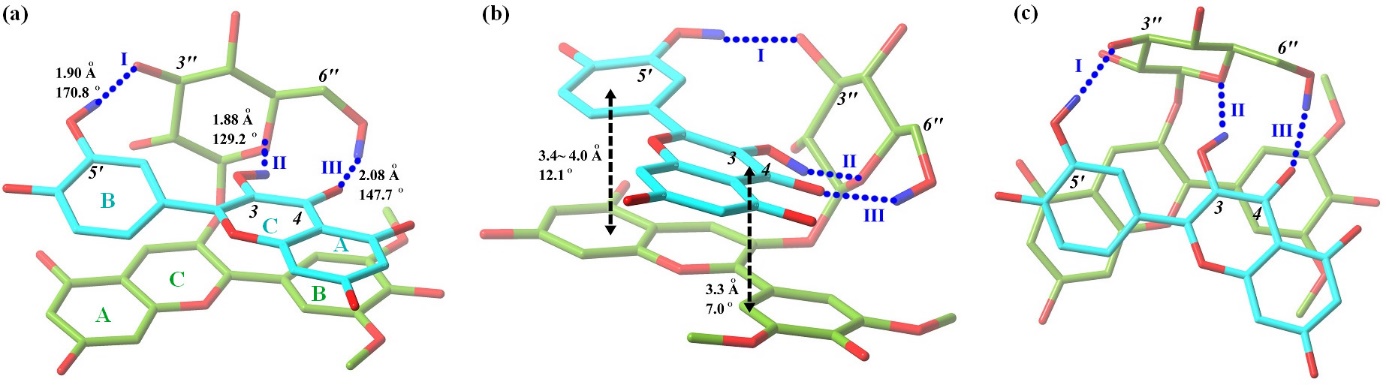


**Figure S2-7**. Front (a), side (b) and top (c) views of conformer **8**


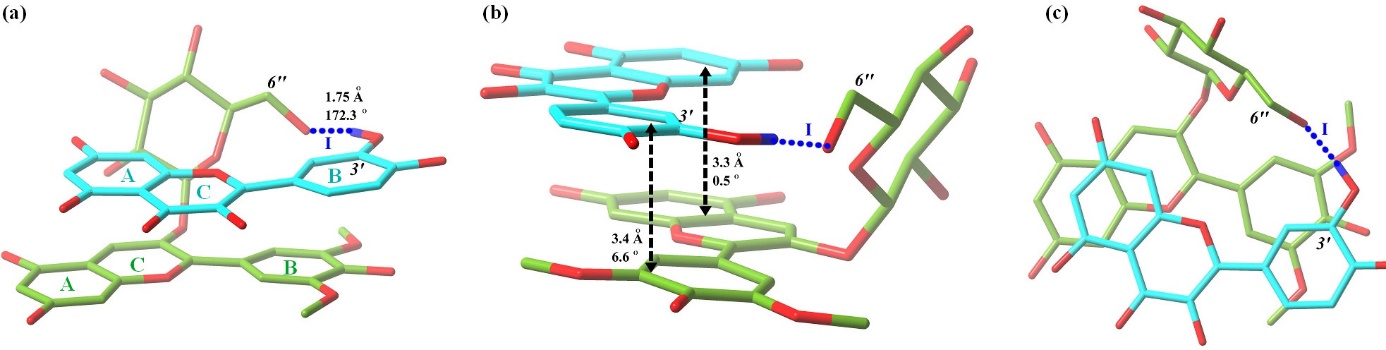


**Figure S2-8**. Front (a), side (b) and top (c) views of conformer **9**


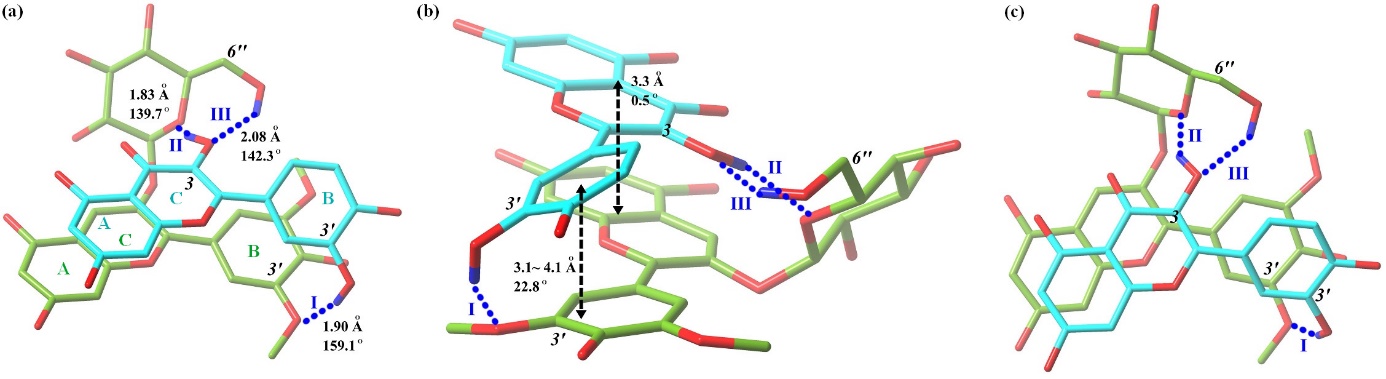


**Figure S2-9**. Front (a), side (b) and top (c) views of conformer **10**


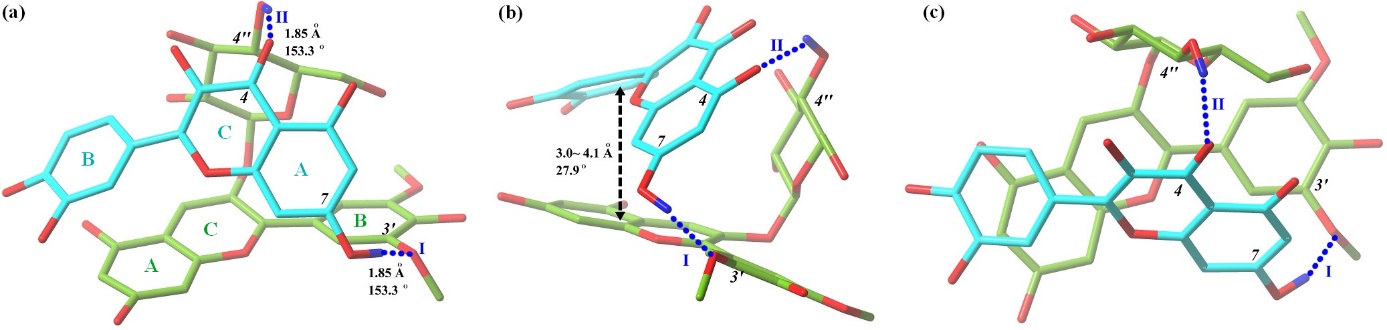


**Figure S2-10**. Front (a), side (b) and top (c) views of conformer **11**


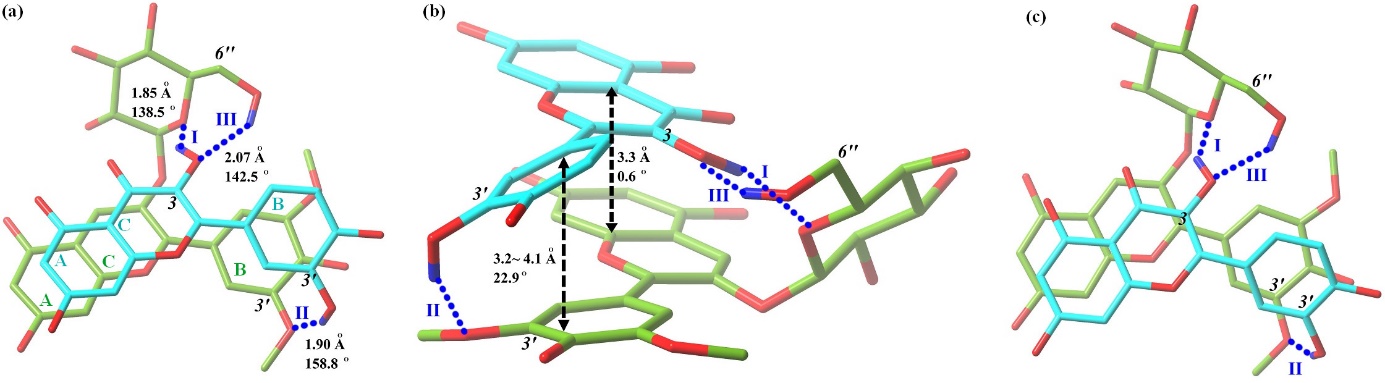


**Figure S2-11**. Front (a), side (b) and top (c) views of conformer **12**
